# Epidemiology and Laboratory Diagnostics of Dengue, Yellow Fever, Zika, and Chikungunya Virus Infections in Africa

**DOI:** 10.3390/pathogens10101324

**Published:** 2021-10-14

**Authors:** Awadalkareem Adam, Christian Jassoy

**Affiliations:** Institute for Medical Microbiology and Virology, University Hospital and Medical Faculty, University of Leipzig, Johannisallee 30, 04103 Leipzig, Germany

**Keywords:** dengue virus, Zika virus, yellow fever virus, chikungunya virus, epidemiology, laboratory diagnostics, Africa

## Abstract

Arbovirus infections are widespread, and their disease burden has increased in the past decade. In Africa, arbovirus infections and fever with unknown etiology are common. Due to the lack of well-established epidemiologic surveillance systems and accurate differential diagnosis in most African countries, little is known about the prevalence of human arbovirus infections in Africa. The aim of this review is to summarize the available epidemiological data and diagnostic laboratory tools of infections with dengue, yellow fever, Zika, and chikungunya viruses, all transmitted by Aedes mosquitoes. Studies indicate that these arboviral infections are endemic in most of Africa. Surveillance of the incidence and prevalence of the infections would enable medical doctors to improve the diagnostic accuracy in patients with typical symptoms. If possible, arboviral diagnostic tests should be added to the routine healthcare systems. Healthcare providers should be informed about the prevalent arboviral diseases to identify possible cases.

## 1. Introduction

Arboviruses are an expanding public health threat in endemic areas. Outbreaks of arboviral infections have occurred worldwide, particularly in tropical, subtropical, and developing countries. Outbreaks of disease regularly raise public concern and challenge the health system and politics of the affected countries alike.

Arboviruses are a group of viruses that are transmitted via arthropods, such as mosquitoes, sand flies, and ticks [1]. Families within the arboviruses that cause disease in humans are Flaviviridae, Togaviridae, Bunyaviridae, and Reoviridae [2]. Within these families, yellow fever virus (YFV), dengue virus (DENV), Zika virus (ZIKV), West Nile virus (WNV) (Flaviviridae), chikungunya virus (CHIKV), O’nyong-nyong virus (ONNV) (Togaviridae), Rift Valley fever virus (RVFV), and Crimean–Congo hemorrhagic fever virus (CCHFV) (Bunyaviridae) are medically important because of the disease burden, severity of disease, and unexpected outbreaks.

Infections with arboviruses depend on the availability and distribution of arthropods. WNV is transmitted by Culex mosquitoes [3,4] and has caused sporadic outbreaks [5]. Transmission of WNV between humans is unlikely because of the low viral concentrations in human blood. ONNV is transmitted by mosquitoes of the genus Anopheles and human vector–human transmission occasionally causes large disease outbreaks [6,7]. CCHFV is transmitted by ticks and by direct contact with the blood of infected patients [8,9]. It causes sporadic disease and small nosocomial outbreaks [8]. RVFV is transmitted between animals by Aedes mosquitoes [10,11]. It causes large outbreaks in livestock animals that subsequently affect humans that are in close contact with the animals. Transmission to humans occurs by direct contact with body fluids and aerosols from infected cattle and by mosquito bites. DENV, YFV, ZIKV, and CHIKV are transmitted from human to human by the same mosquito vectors. The primary vector of these viruses in Africa is *Aedes aegypti* [12,13]. *Aedes albopictus*, which was introduced into Africa less than 30 years ago, increasingly contributes to transmission of these viruses [14].

Clinical symptoms of infections with arboviruses overlap with other febrile illnesses. Therefore, laboratory confirmation is required for correct diagnosis. Acute infection can be diagnosed molecularly by detecting viral RNA using reverse transcription polymerase chain reaction (RT-PCR) and other nucleic acid amplification tests, or by antigen tests. In addition, virus-specific IgM antibodies are generally a marker of acute infection. In some African countries, few clinics have the equipment, expertise, and financial means for laboratory testing for arboviral infections. Thus, medical doctors rely heavily on clinical experience and judgement. Clinical assessment of disease uses epidemiological information about the likelihood of a disease in a particular situation. A high frequency of an infectious disease in a particular region raises the probability that a patient with characteristic symptoms has the disease. For instance, malaria is frequent in tropical Africa, and the disease is an important differential diagnosis in a patient with acute fever. In contrast, malaria is improbable in a patient in the U.S. or Germany unless the person has traveled to a malaria-endemic country. Relating to arboviral infections, information about the distribution of the infections in a particular region allows doctors to correctly include or exclude these infections in the differential diagnosis.

Evidence indicates that DENV, YFV, ZIKV, and CHIKV are endemic in Africa.

Since there are no well-established epidemiological surveillance systems and laboratory diagnosis, there is a lack of information about the incidence and prevalence of infection with these viruses in Africa. On the other hand, the WHO regularly reports about outbreaks with arboviruses and other infectious agents [15]. Information about the distribution and spread of arbovirus infections is important for prophylactic measures. The most potent prophylactic measure is vaccination. Currently, there are commercially available vaccines for yellow fever (commercial names YF-Vax and Stamaril) [16] and for DENV (Dengvaxia) [17]. However, there are no commercially available vaccines to prevent ZIKV or CHIKV infections, although there are multiple vaccine platforms under development [18,19,20]. The dengue vaccine was registered in several countries for use in individuals 9–45 years of age [21]. In Africa, the vaccine has been approved in South Africa where DENV is not endemic. The astonishing development of several potent vaccines against SARS coronavirus-2 in less than a year raises hopes that new vaccine technologies will speed up the development of vaccines against some of the arboviral diseases, as well.

## 2. Distribution of *Aedes aegypti* and *Aedes albopictus* in Africa

**Figure 1:**
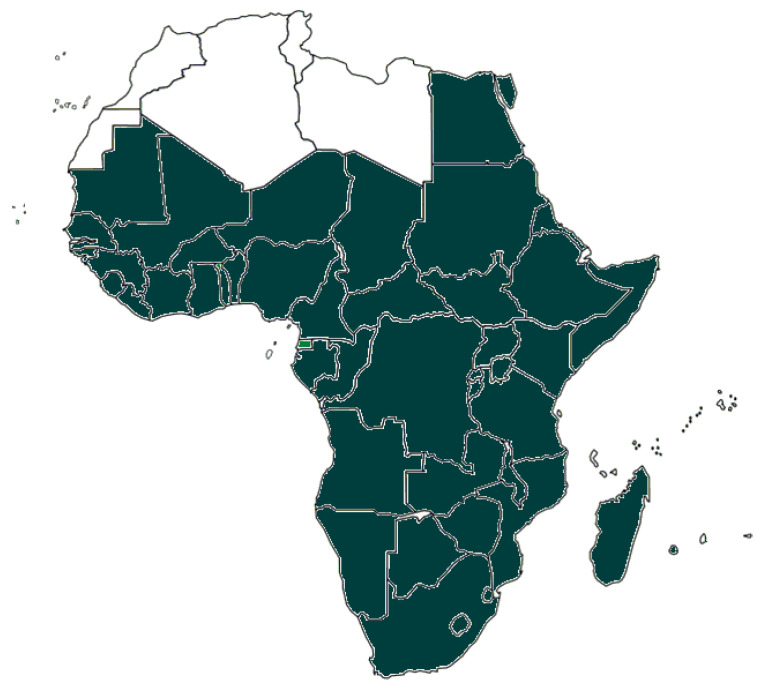
African countries having reported the presence of *Aedes aegypti.* Presence of the mosquito species may be restricted to parts of the countries.

**Figure 2:**
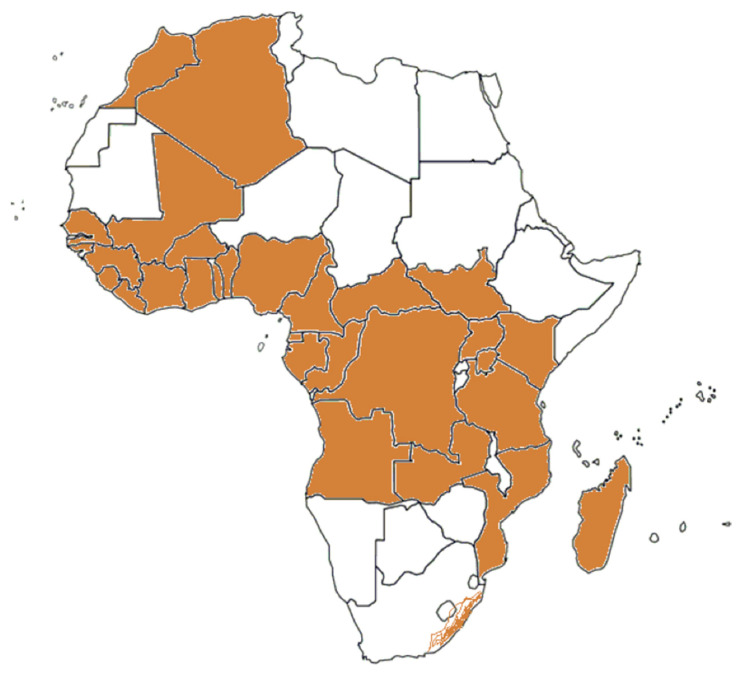
The geographic distribution of *A. albopictus* in Africa.

The mosquito *Aedes* (*Stegomyia*) *aegypti* (L.) and the mosquito *Aedes albopictus* (Skuse) are two major vectors for several arboviruses [22]. *Aedes aegypti*, in particular, originated in sub-Saharan Africa from a wild and zoophilic ancestral species named *A. aegypti* formosus [23]. Due to globalization and different human activities, *A. aegypti* has been introduced and established in tropical and subtropical regions worldwide [22]. *Aedes aegypti* feeds almost exclusively on humans during daylight and rests indoors [24]. It is considered to be the primary vector of YFV, DENV, ZIKV, and CHIKV. In addition, several other African Aedes species are competent and epidemiologically significant vectors. *A. aegypti* has been reported from most African countries (Figure 1). Inhabitants in the *A. aegypti* areas are potentially exposed to DENV, YFV, ZIKV, and CHIKV by mosquito bites. Therefore, *A. aegypti* areas can be considered as endemic regions and/or regions at risk for infections by these viruses. *Aedes albopictus* is also known as the Asian tiger mosquito because of its geographic origin and the black and white stripes on its body and legs. It is native to Southeast Asia, islands of the Indian Ocean, and the Western Pacific [24,25]. It later expanded to Africa, Europe, and the Americas via human activities and transportation [25,26]. *A. albopictus* alternatively feeds on humans and animals and tends to rest outdoors [27]. The geographic distribution of *A. albopictus* is restricted to some areas in Africa (Figure 2).

## 3. DENV Infections

DENV causes a wide spectrum of clinical manifestations which range from asymptomatic infection and dengue fever to severe dengue. Dengue fever is characterized by fever, nausea, vomiting, rash, aches and pains behind the eyes, and muscle, joint, or bone pain. Symptoms of dengue fever usually last for 2–7 days. Warning signs of severe dengue appear in the 24–48 h after the fever has gone away. They include feeling tired, restless, or irritable, belly pain, tenderness, repeated vomiting (at least three times in 24 h), bleeding from the nose or gums, vomiting of blood, or blood in the stool [28].

DENV belongs to the genus Flavivirus within the *Flaviviridae* family [29]. There are four antigenically related DENVs which are DENV-1, DENV-2, DENV-3, and DENV-4 [30]. The origin of DENV is unknown. The first possible dengue outbreak was reported around the 1780s in Philadelphia, USA [31]. Today, DENV is common in more than 100 countries around the world. It is the most prevalent viral infection transmitted by mosquitoes, with one model estimating about 390 million cases annually in tropical and subtropical regions [32].

**Figure 3:**
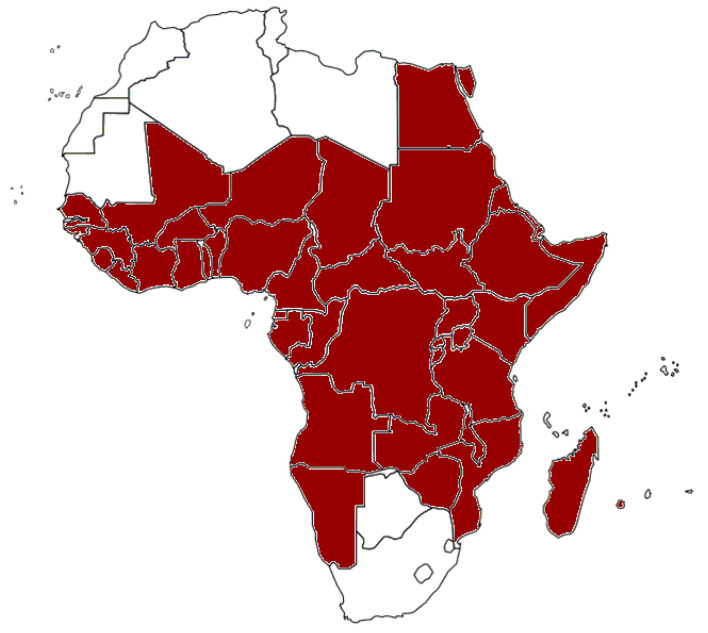
African countries from which cases of dengue fever have been reported.

In Africa, the first officially recognized dengue outbreaks were reported in Zanzibar in 1823 and 1870 [33]. In the early 20th century, outbreaks of dengue fever were reported from South Africa, Egypt, Senegal, and Burkina Faso. By 2020, sporadic cases or outbreaks had been reported in 46 countries in Africa. These countries include Burundi, Comoros, Djibouti, Eritrea, Ethiopia, Kenya, Madagascar, Malawi, Mauritius, Réunion, Rwanda, Seychelles, Somalia, Tanzania, Uganda, Sudan, and South Sudan (East Africa); Benin, Burkina Faso, Cape Verde, Côte D’Ivoire, Gambia, Ghana, Guinea, Guinea-Bissau, Liberia, Mali, Mauritania, Niger, Nigeria, Senegal, Sierra Leone, and Togo (West Africa); Cameroon, Central African Republic, Chad, Congo, Democratic Republic of the Congo, Equatorial Guinea, and Gabon (Central Africa); Angola, Mozambique, Namibia, Zambia, and Zimbabwe (Southern Africa); and Egypt (North Africa) [34,35,36] (Figure 3 and Table 1). All four DENV serotypes have been reported in Africa. In a recent meta-analysis, the overall seroprevalence of DENV in healthy individuals was 15.6% (95% confidence interval 9.9–22.2%) [37]. The seroprevalence of dengue in different regions on the African continent was mostly below 40%, but seroprevalence studies from Mali, Nigeria, Sierra Leone, and Sudan reported regional IgG frequencies of more than 70% [36,38,39,40,41,42].

## 4. Yellow Fever

Yellow fever (YF) is found in tropical areas of Africa and South America. The virus likely originated in Africa and was imported into America during colonization possibly on board of slave trading sailing vessels [107]. In Africa, YFV is transmitted between humans by *A. aegypti* and other mosquito vectors as a domestic/peri-domestic disease [108]. Many infections with YFV are asymptomatic or mild. Symptoms include sudden fever, chills, headache, low back pain, myalgia, nausea, vomiting, fatigue, hepatitis with jaundice, hemorrhage and shock with renal failure, and multisystem organ failure. About 15% of infected people develop severe visceral disease. The fatality among patients with severe disease is 20–60% [109].

The virus is zoonotic and infects monkeys as natural hosts. In Africa, most monkey species are poorly susceptible to disease. When they become infected, they are asymptomatic or develop mild symptoms but rarely develop fatal disease, and they later become immune. In contrast, monkeys in the New World are frequently affected by severe disease and die.

A highly effective live-attenuated vaccine based on the African YFV isolate Asibi and named YFV-17D was developed in the 1930s [110,111]. The vaccine was produced in embryonated eggs and has been used for about 80 years. There are three 17D sub-strains, namely, 17D-204, 17DD, and 17D-213, that are used as vaccines, which have minor differences in the genome sequence [110,112]. In 1941, YF vaccination became mandatory in French colonies in Africa and contributed to the effective control of YFV infection. In Africa, childhood YFV vaccination is common and still expanding. YF vaccination is highly recommended or mandatory for travelers to Central, West, and East African countries or areas with risk of virus transmission.

**Figure 4:**
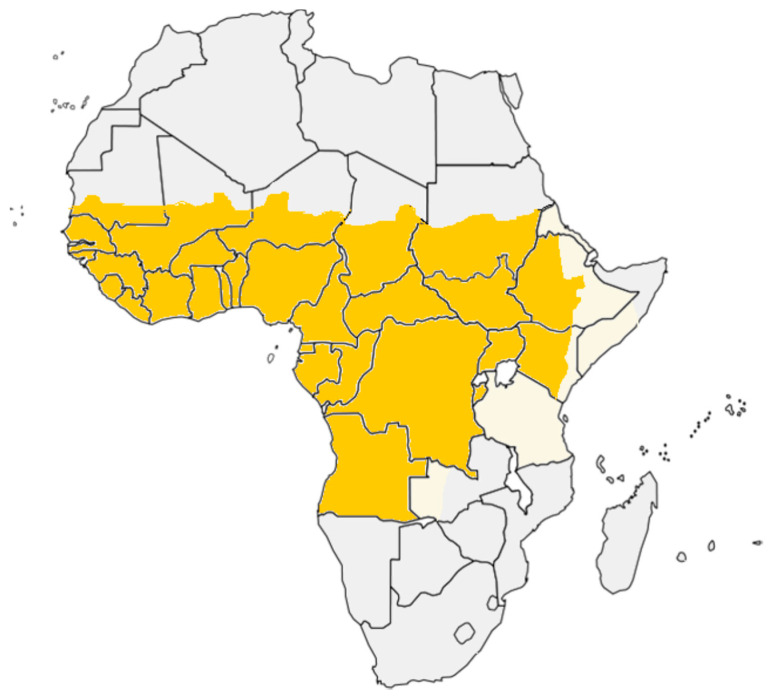
Countries and regions from which yellow fever cases have been reported.

Sporadic YF cases and small epidemics have been reported in West Africa since the end of the 15th century. However, due to the lack of an appropriate vector at that time, the disease was absent in East and South Africa. In the period between the 1960s and 1980s, YF outbreaks were reported in Central and West Africa [113]. In the period between 1960 and 1962, YF epidemics were reported from Ethiopia, with around 100,000 cases and 30,000 deaths [88,114]. In the period between 1984 and 1990, a YF epidemic was reported from Nigeria and associated with 21,299 deaths [115]. Since the late 1990s, several outbreaks of YF have occurred throughout sub-Saharan countries. At present, serologic evidence or outbreaks of YFV infections or both have been reported in 29 African countries. These countries include Angola, Benin, Burundi, Burkina Faso, Cameroon, Central African Republic, Chad, Cote d’Ivoire, Democratic Republic of the Congo, Ethiopia, Equatorial Guinea, Gabon, Gambia, Ghana, Guinea, Guinea-Bissau, Kenya, Liberia, Mali, Mauritania, Nigeria, Niger, Rwanda, Senegal, Sierra Leone, South Sudan, Sudan, Togo, and Uganda (Figure 4 and Table 1).

## 5. Zika Virus Infections

ZIKV has recently emerged as a clinically important virus. Infections are usually asymptomatic. However, in some cases, there are mild symptoms which include fever, rash, headache, arthralgia, and conjunctivitis [116,117]. In adults, ZIKV infection has been linked to Guillain–Barré syndrome (GBS), neuropathy, and myelitis [118]. Severe cases are uncommon, and fatalities are rare. An outbreak of ZIKV in Brazil in 2015 was associated with microcephaly in infants born from infected women and novel patterns of direct virus transmission via sexual intercourse [119,120,121].

ZIKV is closely related to DENV. The virus has two major genotypes known as the African and Asian lineages [122]. ZIKV was discovered in 1947 in the Zika forest, Uganda. It was isolated from the blood of a sentinel rhesus monkey during YFV surveillance [123]. One year later, ZIKV was isolated from *Aedes africanus* mosquitoes collected in the same forest [123]. In 1952, the first evidence of human infection was reported by detecting neutralizing antibodies in human sera from East Africa. A ZIKV outbreak occurred on Yap Island, Federated States of Micronesia, in 2008, and the virus caused a large outbreak in most of Latin America in 2015. Today, ZIKV remains a threat to global public health.

**Figure 5:**
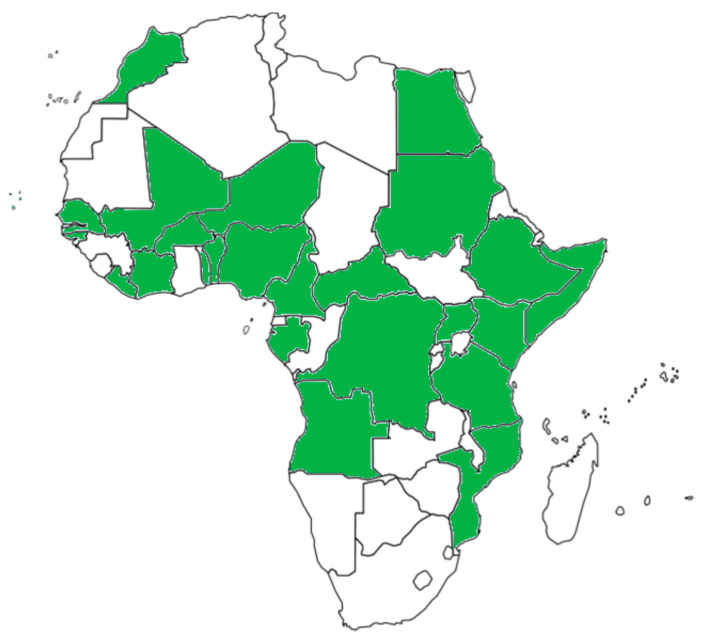
African countries from which ZIKV cases or serologic evidence of ZIKV infections have been reported.

In Africa, molecular and serological evidence of ZIKV has been reported from 26 countries. These include Angola, Benin, Burkina Faso, Cabo Verde Islands, Cameroon, Central African Republic, Côte d’Ivoire, Democratic Republic of the Congo, Ethiopia, Egypt, Gabon, Guinea-Bissau, Kenya, Mali, Morocco, Mozambique, Nigeria, Niger, Senegal, Somalia, Sudan, Tanzania, Togo, and Uganda (Figure 5). The evidence for ZIKV infections in these countries is summarized in Table 2.

## 6. Chikungunya Fever

The name “chikungunya” comes from the Tanzanian Makonde dialect which means “that bend up the joint”. Most people infected with CHIKV have fever, mild to incapacitating joint pain, swelling of the joints, headache, myalgia, maculopapular rash, and nausea. Some patients also develop severe joint pain [147,148]. Joint pains and myalgia can persist for several months, particularly in females and in the elderly [149,150,151]. A total of 2–25% of CHIKV-infected individuals are asymptomatic. CHIKV is considered to cause a higher rate of symptomatic infections compared to DENV and ZIKV.

**Figure 6:**
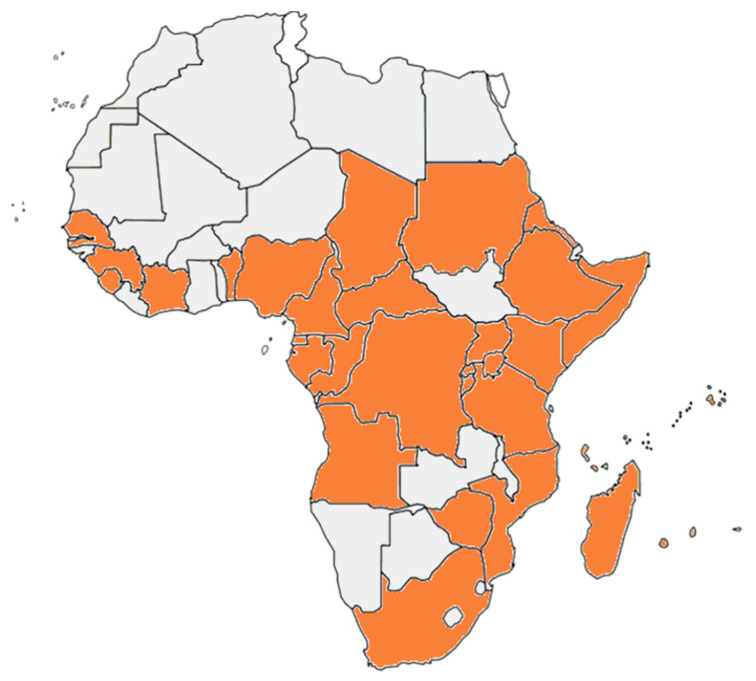
African countries from which chikungunya outbreaks or serologic evidence of the infection have been reported.

CHIKV is an Alphavirus within the family Togaviridae which also includes O’nyong-nyong virus (ONNV) and Semliki Forest virus [152]. These viruses have a single-stranded positive-sense RNA genome [152]. CHIKV is transmitted to humans by mosquitoes of the genus Aedes, particularly *Aedes aegypti* and *Aedes albopictus* [153,154,155]. The virus was first isolated from blood samples collected during an outbreak in Tanzania in 1952 [156,157]. It is likely that the virus caused disease outbreaks before, but the disease was not recognized. Since then, three genotypes of the virus have been recognized. These genotypes are named East/Central/South African (ECSA), West African, and Asian [158]. In Africa, CHIKV is maintained in a sylvatic transmission cycle, as with YFV, involving non-human primates and *Aedes africanus* and *A. furifer* mosquitoes, and an urban cycle with the virus being transmitted by *Aedes aegypti* and *A. albopictus* [153,159]. In rural areas, the virus is endemic and causes sporadic infections. In urban environments, the virus causes massive outbreaks that infect a large percentage of the population in several weeks. CHIKV caused multiple outbreaks in Africa. The virus has been detected in humans and mosquitoes during outbreaks. In addition, seroprevalence studies have been performed in several African countries. In the period from the first CHIKV outbreak in Tanzania in 1952–1953 until now, evidence of CHIKV infections and serologic evidence of previous infection have been reported from 33 African countries. These countries include Angola, Benin, Burundi, Cameroon, Central African Republic, Chad, Comoros, Cote d’Ivoire, Democratic Republic of the Congo, Djibouti, Equatorial Guinea, Eritrea, Ethiopia, Gabon, Guinea, Kenya, Madagascar, Malawi, Mauritius, Mayotte, Mozambique, Nigeria, Republic of the Congo, Réunion, Senegal, Seychelles, Sierra Leone, South Africa, Somalia, Sudan, Tanzania, Uganda, and Zimbabwe [160,161,162] (Figure 6). During the period from 2004 to 2020, CHIKV was reported in Kenya, Comoros, La Réunion, Mauritius, Seychelles, Madagascar, Mayotte, Cameroon, Tanzania, Gabon, Republic of the Congo, Senegal, and Angola [94,96,98,102,103,163,164,165,166,167,168]. Despite CHIKV being discovered in East Africa seventy years ago, there is still a lack of data on the prevalence of the infection and the disease burden in Africa.

## 7. Laboratory Diagnosis of Arboviral Infections in Africa

### 7.1. Diagnostic Challenges for Arboviral Infections

In Africa, the main challenge is differentiating between etiologies of febrile illnesses with similar initial clinical presentations, including malaria, influenza, measles, dengue fever, YF, chikungunya fever, acute HIV infection, Lassa fever, typhoid fever, bacterial meningitis, leptospirosis, and other infections. Therefore, misdiagnosis of arboviral infections and confusion with other etiologies are common. As there is no specific treatment for arboviral infections and treatment mainly focuses on supportive care, the main reasons to diagnose the infection correctly are to exclude other treatable diseases, and to early identify regional clusters of infection. In addition, correct laboratory diagnosis provides important feedback to doctors and healthcare workers to raise medical quality and self-efficacy. Laboratory methods for diagnosis include detection of the viral genome, viral antigen, and IgM antibodies.

### 7.2. Diagnosis of Acute and Previous Infection with Arboviruses

Several approaches can be used for the laboratory diagnosis of acute DENV, ZIKV, and YFV infection. Viremia and antigenemia typically occur between 2 and 3 days before the onset of fever and last for 2–7 days of illness. Thus, acute infection can be confirmed by detection of the viral genome or, in the case of DENV infection, by NS1 antigen tests. IgM examination can be used beginning 4–5 days after the onset of illness [169].

Previous infections can be confirmed in the laboratory by serological tests that examine IgG antibodies. These tests are used primarily for seroprevalence studies and to assess the level of protection from infection in a population. Serological methods such as enzyme-linked immunosorbent assays (ELISAs), immunofluorescence tests (IFTs), rapid diagnostic tests (RDTs), and virus neutralization tests (NTs) can be used for the detection of antibodies against DENV, ZIKV, and YFV.

Sera from individuals infected with different flaviviruses frequently cross-react in antibody tests. Cross-reactivity is due to amino acid sequence similarities between homologous proteins of different flaviviruses, especially DENV and ZIKV [170,171,172,173]. Serum from a person who has been infected with DENV may react when tested with a test for antibodies against ZIKV or West Nile virus. Therefore, serological tests for these viral infections lack specificity, particularly in individuals who have had previous flavivirus exposure. Neutralization tests using live viruses are considered the gold standard tests for serological analysis of previous flavivirus infections [174]. Neutralization tests require culturing viruses and must be performed in a high biosafety level facility. Although these assays offer greater specificity compared with ELISAs, cross-reactions occur frequently between DENV, ZIKV, and West Nile virus [175]. In our own studies, we have not seen cross-reaction of sera from YFV-vaccinated individuals with DENV shortly after vaccination, indicating that DENV neutralization assays are not affected by YFV vaccination (unpublished observation).

Chikungunya virus can be diagnosed by RT-PCR and IgM antibody ELISA. Serological assays for CHIKV do not cross-react with serological assays for DENV, ZIKV, or YFV. Detection of CHIKV IgM and IgG antibodies using ELISA is relatively cheap and easy to perform, but in general, CHIKV tests are not part of routine laboratory tests in African countries.

### 7.3. Laboratory Testing during Arboviral Disease Outbreaks

Routine arbovirus diagnostics are rarely performed by medical systems in the majority of African countries due to the higher importance of diagnosis of malaria, HIV, and tuberculosis. In addition, the latter infections can be treated with antimicrobial and antiviral agents. However, during times of arbovirus outbreaks, there is a shift towards arbovirus testing. When arbovirus outbreaks occur in rural areas with limited laboratory facilities for diagnosis, local clinics contact the responsible public health institutions of the ministry of health to receive a team of experts to investigate the outbreak and collect samples. The samples are to be sent to the capital city for laboratory testing. Due to difficulties in transportation in rural areas, there is frequently a delay in the shipment of samples, resulting in a decrease in sample quality. Shipment and detection of samples are constrained even more when arbovirus outbreaks occur within nomadic communities. Recently, mobile laboratories able to perform recombinase polymerase amplification (RPA) assays have been used for diagnosis of arbovirus infections in low-resource settings [176].

Due to the limited resources/facilities in many African countries [177], RT-PCR and other genome detection systems for the diagnosis of flaviviruses and CHIKV are not common as of yet. However, countries such as South Africa, Cote d’Ivoire, and Senegal (Institute Pasteur), as well as Nigeria, have well-established laboratories to perform the molecular diagnosis of arboviruses and have started to include flaviviruses and CHIKV diagnoses in routine tests. However, countries such as Sudan, Egypt, Ethiopia, and Kenya do not include RT-PCR for the diagnosis of arboviruses in outpatient routine tests. However, during outbreaks and research studies, RT-PCR is requested and performed in national laboratories. Other countries in Africa do not have the facilities and trained staff to perform molecular tests; therefore, they rely on sending the samples to established laboratories in other African countries or Europe.

Rapid antibody tests are available in most African countries and are used during outbreaks as well as in laboratories with limited technical facilities. Antibody tests, as with ELISAs for flaviviruses and CHIKV, are performed during outbreaks or in suspected cases that may indicate an outbreak, but are still not included as a routine test in the majority of African countries. Moreover, neutralization assays for flaviviruses are feasible and are performed in limited African countries such as South Africa and Senegal; however, they are still not included in routine tests.

## 8. Discussion

### 8.1. Challenges and Chances

Diseases caused by arboviruses can progress to long-term physical consequences and lead to death in some cases. Arboviral diseases are probably common causes of severe febrile illness in the African continent, and a large number of people are at risk for arbovirus infection. Due to the limitations in medical resources in most African countries, routine laboratory testing of arboviruses is not common. Thus, the incidence of the infections is largely unknown. Information about the prevalence of DENV, ZIKV, YFV, and CHIKV will help clinicians in establishing the right diagnosis for these viruses. Current developments in vaccine technology raise hopes that more vaccines for arboviral diseases will be developed in the near future. National laboratories and public hospitals in the capital and some big cities in most African countries are reasonably well equipped for laboratory diagnosis of viral infections. These institutions have motivated and good-quality laboratory personnel. Many of these institutions have people being trained in arboviral disease diagnostics and epidemiology abroad and at home and are in collaborative efforts with partner institutions in Europe and the U.S. In some African countries such as Sudan, the private medical sector has made tremendous improvements in the diagnosis of infectious diseases. However, most African countries have limited laboratory facilities and access to reagents, both of which would affect the accuracy of diagnosis of acute arbovirus infections. In addition to that, most African countries do not have high biosafety level facilities to perform virus isolation and neutralization tests. These countries rely on IgM and IgG antibody ELISAs and send samples to more established systems in other African countries, to the USA, or to Europe for further confirmation.

### 8.2. Future Perspective for Epidemiological Monitoring

Few data exist about the incidence and prevalence of arbovirus infections in Africa; therefore, little is known about the potential impact of arboviruses on the health of the population. Although some seroprevalence studies have been conducted to estimate the spread of arboviruses in Africa, there are several limitations to these studies. The sample size of these studies is generally low. Additionally, most of the studies that examined the prevalence of infections with DENV, ZIKV, and YFV used ELISAs to detect IgG antibodies. Flaviviruses share common epitopes which induce cross-reactive antibodies [170]. Cross-reactive antibodies lead to great difficulty in differential sero-diagnosis of flavivirus infections. Some of these studies used neutralization assays for confirmation, but a fraction of sera from acutely or previously infected individuals cross-react in these assays. In particular, the ZIKV neutralization assay can significantly cross-react with DENV antibodies. YFV neutralization tests differentiate YFV infection and previous YFV vaccination from infection by other flaviviruses. Antibody tests for CHIKV reliably differentiate from flavivirus infections but cross-react with sera after O’nyong-nyong virus infection. Lastly, the majority of the seroepidemiological studies are older, some even older than 30 years, which makes the data not applicable to the current epidemiological situation. Therefore, solutions include conducting studies with larger sample sizes and using specific detection methods. Additionally, it would be beneficial to establish or strengthen national and regional arbovirus surveillance systems in Africa.

Multiple recent arbovirus outbreaks have been described in different parts of Africa. It is possible that many small outbreaks occurred in the nomadic community and remained unrecognized because of their local migrations or due to the very limited capacities for detection and monitoring of the outbreaks in most of the continent. The high prevalence of malaria and other infectious diseases in Africa reduces the attention towards arbovirus infections. Diagnostic testing in the routine healthcare systems and implementing prevention and control policies in the general population should attain higher priority by healthcare providers, researchers, and health policy makers in Africa. A comprehensive surveillance would help to better understand the burden of arboviral infections, improve clinical diagnostic accuracy, and guide future vaccine implementation when vaccines against DENV, ZIKV, and CHIKV become available.

### 8.3. Priorities

Diagnostic and surveillance systems must be further developed in the public health systems. Diagnostic and surveillance systems can be improved through training of more African laboratory scientists and technicians on how to collect the right samples, perform accurate and reliable laboratory tests, and correctly interpret the data. This training should be considered a priority and, if done on the spot, requires adequate laboratory equipment and testing capabilities. Establishing decentralized well-equipped laboratories in rural areas and more central laboratories around the continent is also important to increase the accessibility of testing. From there, the next step would be to perform screenings of the population to determine the extent of past arboviral infections and draw a detailed and updated arboviral infection map of Africa. Experience shows that it is difficult to obtain blood samples for surveillance testing. The optimal sample for seroepidemiologic studies is random selection of patients coming to outpatient clinics and plasma from blood banks. The general population, patients, and blood donors should be educated about arboviral diseases and the benefit of serosurveillance in order for them to consent to testing. Physicians should be aware of arboviral symptoms in order to reach the appropriate clinical diagnosis. Tracking community outbreaks in cities and rural areas and detecting arboviruses that are currently circulating are also urgently needed. This can be achieved through viral RNA detection and, ideally, through viral isolation. Statisticians and epidemiologists are also urgently needed to collect data, conduct documentation, and perform statistical analyses. In order to implement these systems, there needs to be increased funding from governments. Investments will be rewarding because they will improve people’s health and increase medical technology, knowledge, and expertise in these countries.

## Figures and Tables

**Table 1:** Significant outbreaks of dengue fever, yellow fever, Zika, and chikungunya virus infections in Africa since the year 2000.

Virus	Country	Years	Reference
**Dengue fever**	Sudan	2004, 2015	[42,43]
	Kenya	2011, 2013, 2014, 2017	[44,45,46]
	Burkina Faso	2016, 2017	[47,48]
	Mozambique	2014	[49]
	Angola	2013	[50,51]
	Tanzania	2014, 2018–2019	[52,53,54]
	Gabon	2007, 2010	[55]
	Senegal	2009, 2017, 2018–2020	[56,57,58]
	Cote d’Ivoire	2017, 2019	[54,59]
	Seychelles	2015, 2020	[15,58]
	Benin	2019	[54]
	Ethiopia	2018, 2019	[15]
	Mauritius	2019	[60]
	Kenya	2018–2019, 2021	[15,61]
	Mauretania	2018	[58]
	Mali	2019–2020	[15]
**Yellow fever**	Nigeria	2018, 2020, 2021	[54,62,63,64]
	Sudan	2011, 2012, 2018	[65,66,67,68]
	Côte d’Ivoire	2001–2003, 2006, 2010, 2011, 2019	[54,69,70,71]
	Senegal	2020	[72]
	Guinea	2000–2001, 2008, 2009	[73,74,75]
	Liberia	2004	[76]
	Cameroon	2017-2021	[77]
	Ghana	2012	[78]
	Sierra Leone	2011	[79]
	Republic of South Sudan	2003	[80]
	Togo	2006	[81]
	Central African Republic	2009	[82]
	Uganda	2010, 2011, 2016, 2019	[54,83,84]
	Democratic Republic of the Congo	2015–2016	[85,86,87]
	Ethiopia	2013	[88]
	Angola	2015–2016	[87]
**Zika**	Angola	2017	[89]
	Cabo Verde Islands	2015, 2016	[90,91]
**Chikungunya**	Kenya	2004, 2016, 2018	[92,93,94,95]
	Gabon	2007, 2010	[96,97]
	Democratic Republic of the Congo	2011, 2019–2020	[95,98,99,100]
	Cameroon	2006	[101,102]
	Senegal	2009–2010, 2015	[95,103]
	Sudan	2005, 2018	[66,95,104]
	Ethiopia	2019	[105]
	Sierra Leone	2012	[106]

**Table 2:** Reports of ZIKV detection or serologic evidence of ZIKV infection in African countries.

Country and Region	Participants	Year	Result	Reference
Mozambique	152 adults, 107 children	1957	4% of sera neutralized ZIKV	[124]
Senegal		1990	ZIKV isolated from patient and IgM antibody were also detected	[125]
	211 adults and children	2011–2012	14 positive blood samples, 22.7% IgG-positive, and 13.7% neutralized ZIKV	[126]
	284 adults and children	2007	21.9% Zika IgG-positive, 13.4% neutralized ZIKV	[126]
Guinea-Bissau	15 infants, 15 mothers	2016	14 samples were IgG-positive, 93% neutralized ZIKV	[127]
Burkina Faso		1963–1964	IgG antibodies were detected	[126]
Mali		1964–1967	Antibodies detected in human blood samples	[128]
	291 participants	2007	0.3% of sera neutralized ZIKV	[126]
	1430 healthy volunteers	2013–2016	11.98% were IgG-positive ZIKV	[128]
Benin		1967	Antibodies in human blood samples	[129]
Democratic Republic of the Congo	978 samples	2013–2014	34 (3.5%) reacted with ZIKV in ELISA and one serum (3.2%) neutralized ZIKV	[130]
Central African Republic		1961	Antibodies detected in human blood samples	[129]
Nigeria	2 adults, 1 child	1954	ZIKV was isolated	[131]
	children under 16 years old	1951	44% of 97 had neutralizing antibodies	[132]
		1971–1975	ZIKV isolated from 2 cases, 31% ZIKV IgG-positive, 40% of sera neutralized ZIKV	[133,134]
Cape Verde	7580 cases adults, newborn children	2015–2016	IgM was detected in 15 out of 64 sera; 2 samples were PCR-positive	[91]
Uganda	Adults, children	1952	ZIKV antibodies were detected and ZIKV was isolated	[123]
	182 adults, 63 children	1967	5 samples were positive for ZIKV antibody by hemagglutination inhibition test	[135]
Tanzania		1952	Serological evidence of ZIKV	[136]
Ethiopia	3690 participants	1960	ZIKV antibodies were detected	[137]
Kenya			Serological evidence of ZIKV	[138]
	745 samples	2013	Thirty-four (4.6%) were positive by MAC ELISA and 5 sera had neutralizing antibodies	[139]
	327 plasma samples	2016	Two samples had neutralizing antibodies	[140]
Somalia		1966–1967	Antibodies detected in human blood samples	[141]
Sudan	845 participants	2012	530 or 62.7% IgG-positive by ELISA, one sample had neutralizing antibodies	[142]
Gabon	4312 human sera	2007	5 samples RNA-positive	[143]
		1967–1980	Serological evidence of ZIKV, 14.7% positive	[144]
Cameroon	102 febrile patients		11.4% of the tested sera reacted monotypically with ZIKV	[145]
Angola	54 samples, 76 infants, 24 mothers, 685 participants	2016/2017	4 samples RNA-positive	[89]
Egypt	189 human sera	1954	1 serum sample neutralized ZIKV	[146]
Morocco		1968–1969	Antibodies to ZIKV found in people and birds	[129]
